# Laparoscopic Resection of Central Ovarian Cancer Recurrence in the Pelvis Utilizing a Fluorescent Ureteral Near-Infrared Ray Catheter (NIRC): A Case Report

**DOI:** 10.7759/cureus.78970

**Published:** 2025-02-13

**Authors:** Takashi Natsume, Mayumi Kobayashi-Kato, Hiroshi Yoshida, Yasuhito Tanase, Masaya Uno, Mitsuya Ishikawa

**Affiliations:** 1 Department of Gynecology, National Cancer Center Hospital, Tokyo, JPN; 2 Department of Diagnostic Pathology, National Cancer Center Hospital, Tokyo, JPN

**Keywords:** central recurrence, fluorescent ureteral catheter, laparoscopic resection, near-infrared ray catheter, ovarian cancer

## Abstract

The central recurrence of ovarian cancer at the vaginal stump requires anterior pelvic resection if there is an invasion into the bladder triangle. There is a lack of evidence regarding the efficacy of minimally invasive surgery for recurrent ovarian cancers. Therefore, there are no reports of using a fluorescent ureteral near-infrared ray catheter (NIRC) (Cardinal Health Ltd., Tokyo, Japan) in laparoscopic surgery for recurrent ovarian cancers. We reported a 51-year-old woman who underwent laparoscopic resection for recurrent ovarian cancer located on the posterior wall of the vaginal stump, exposed to the vaginal lumen, and invaded the rectum but not the anterior vaginal wall or bladder. A fluorescent ureteral NIRC for the real-time intraoperative visualization of the ureters allowed laparoscopic resection of the recurrent tumor at the vaginal stump with preservation of the bladder and the ureters.

## Introduction

Recurrence of ovarian cancer occurs in 75% of patients with advanced ovarian cancer within two years [1]. Secondary debulking surgery with complete resection for recurrent ovarian cancer has been reported and recommended to prolong disease-free survival [2,3]. However, there is no evidence regarding the efficacy of minimally invasive surgery (MIS) for recurrent ovarian cancer. Some retrospective studies have reported no problems compared with open surgery [4,5], but laparotomy is still performed in many hospitals.

Complications during laparoscopic surgery sometimes occur. Ureteral injury has been reported to occur in less than 1% during laparoscopic hysterectomy [6,7]. Ureteral catheters have been reported to be useful during laparoscopic surgery and have been reported to be equally effective in avoiding iatrogenic ureteral injury, especially with the use of a fluorescent ureteral near-infrared ray catheter (NIRC; Cardinal Health Ltd., Tokyo, Japan) [8]. In our department, ureteral catheters were not usually used, except in cases where ureteral resection is unavoidable due to tumor entrapment or other reasons. In addition, fluorescent ureteral NIRCs were not used to avoid iatrogenic ureteral injury. We used a fluorescent ureteral NIRC to dissect the ureters away from the recurrent tumors and to evaluate whether the tumors invaded the ureters. Laparoscopy is considered useful for the observation of cancer spreads [9]. Therefore, we decided to start laparoscopic observation of a central recurrence of a vaginal stump. If there was strong adhesion to the ureters and invasion into the bladder triangle was suspected, we would have decided to convert the surgery to laparotomy as the anterior pelvic resection. There is no report on the usefulness of a fluorescent ureteral NIRC, which is visualized by laparoscopic observation mode with a near-infrared ray for evaluating the possibility of dissection of the ureters from the anterior vaginal wall and the tumor for recurrent ovarian cancer.

This report discusses a case involving laparoscopic resection of a recurrent tumor at the vaginal stump for evaluating the possibility of dissection of the ureters from both the anterior vaginal wall and the tumor, utilizing a fluorescent ureteral NIRC as a guide.

## Case presentation

A 51-year-old woman had undergone a simple hysterectomy, bilateral adnexectomy, pelvic lymph node biopsy, and partial omentectomy for stage IC of ovarian cancer before she was presented to our hospital. The histological pathology was endometrioid carcinoma grade 2.

**Figure 1:**
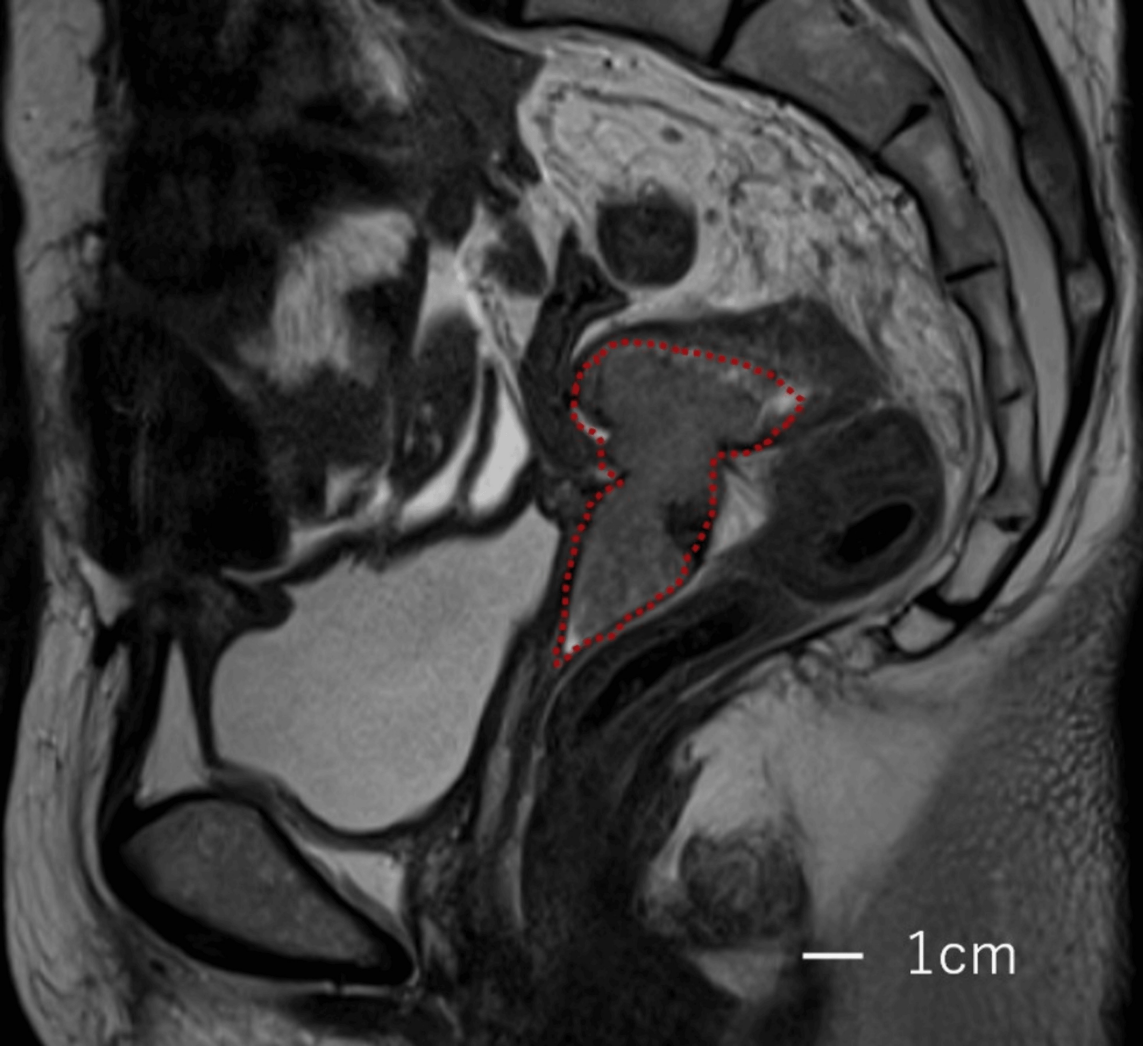
T2-weighted MRI (sagittal), showing the tumor (red dot line) (scale bar = 1 cm) MRI: magnetic resonance imaging

The recurrent tumor was found two years and 10 months after she received conventional paclitaxel and carboplatin therapy (conventional TC therapy) as adjuvant chemotherapy. Then, she received olaparib maintenance therapy after conventional TC therapy as second-line chemotherapy without checking her BRCA mutation status because it is not required for olaparib maintenance therapy for platinum-sensitive recurrent ovarian cancer in Japan. The magnetic resonance imaging showed a recurrent tumor measuring 22 x 25 x 40 mm at the vaginal stump, and there was no invasion to the anterior vaginal wall and the bladder (Figure 1).

**Figure 2:**
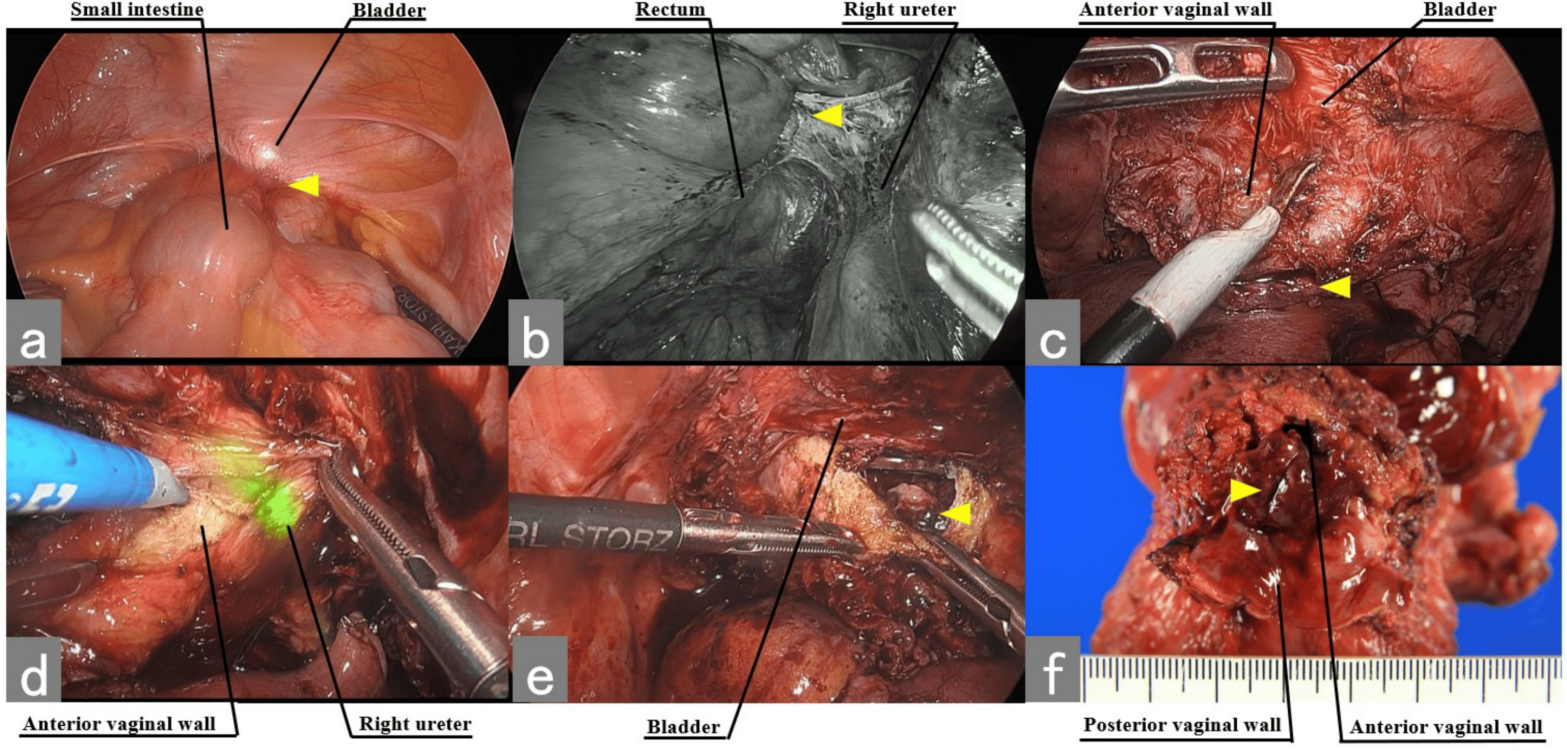
Intraoperative findings: (a) the small intestine was adherent to the recurrent tumor, (b) mobilization of the rectum until the anorectal muscles were exposed, (c) dissection between the bladder and the anterior wall of the vagina to preserve the bladder, (d) dissection between the right urethra and the anterior wall of the vagina to preserve the right urethra when the fluorescent NIRC is visualized, and (e) the anterior wall of the vagina was incised 2 cm away from the tumor. (f) The postoperative specimen The yellow arrowhead shows the tumor

Because no distant metastasis or lymph node metastasis was observed, the patient was referred to our department for surgery. The day before surgery, a fluorescent ureteral NIRC was placed. The intraoperative findings and procedures were as follows (Figure 2).

**Figure 3:**
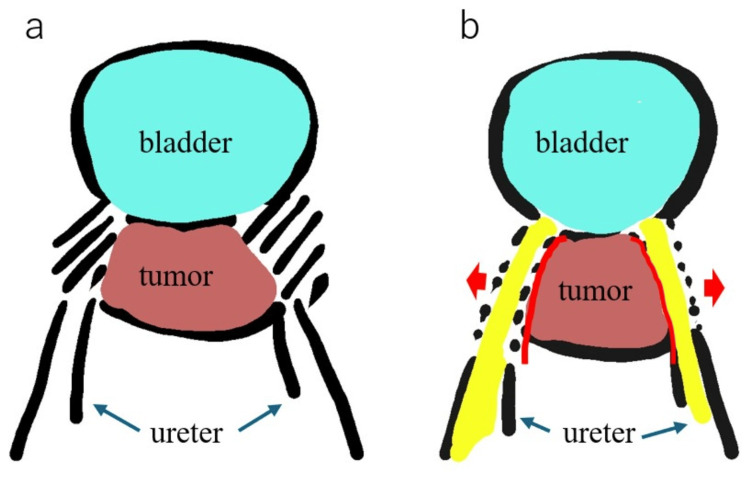
The fluorescent ureteral NIRC under (a) white light and (b) near-infrared rays. (a) The ureter is not clear around the recurrent tumor. (b) The ureter is clear, while the fluorescent ureteral NIRC is visualized. The red lines show the dissection line NIRC: near-infrared ray catheter Image credit: This is an original image created by the authors using Microsoft Paint (Microsoft Corp., Redmond, WA)

There was no seeding or ascites during laparoscopy. The recurrent tumor at the vaginal stump had invaded the small intestine, so the small intestine was partially resected. The first step was to move the rectum. The rectum was moved until the anorectal muscles were exposed. After that, the peritoneum of the vesicouterine pouch was dissected to preserve the bladder and ureters. The running ureters around the tumor were unclear without a fluorescent ureteral NIRC (Figure 3a), and an anterior pelvic resection was needed. However, the NIRC fluorescent ureteral catheter for the real-time intraoperative visualization of the ureters allowed for dissecting the space between the ureters and the tumor and moving the ureter to the outside of the vagina (Figure 3b).

Part of the bladder wall was damaged and later repaired. The spatula was inserted into the vagina to confirm the incision line. The incision line was determined to be 2 cm away from the recurrent tumor. After the incision was determined, the anterior vaginal wall was incised. Then, the posterior vaginal wall was also incised away from the tumor. After suturing the vaginal segment, the rectum was resected. Since the site of partial resection of the small intestine was near the ileocecal region, an additional ileocecal resection was performed.

In this case, the ureters were dissected from the tumor and the posterior vaginal wall due to the real-time intraoperative visualization of the ureters. Then, the bladder and ureter were preserved. The laparoscopic resection of recurrent ovarian cancer, with the tumor invading the rectum and intestines, was successfully performed.

**Figure 4:**
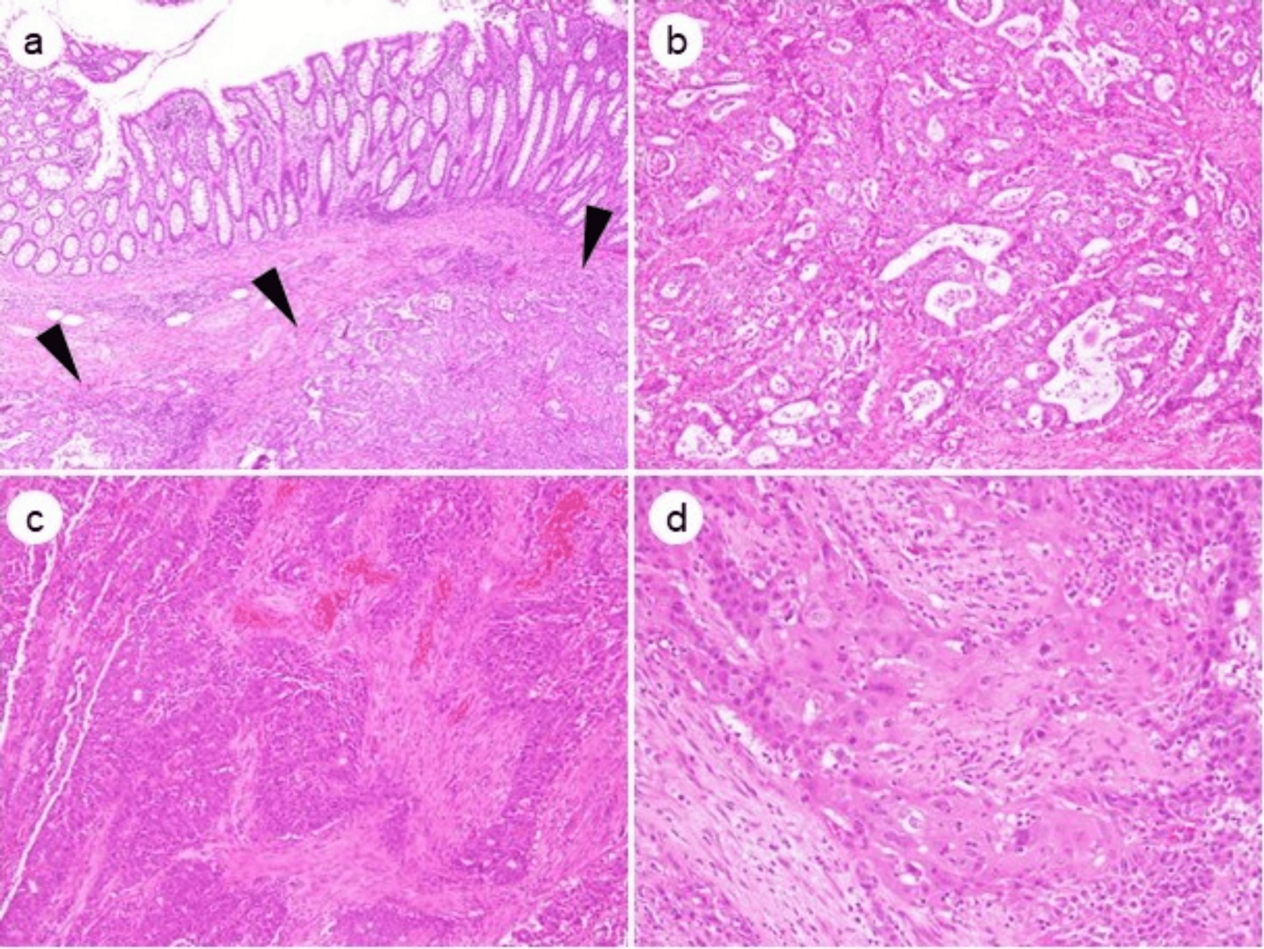
Pathological findings of recurrent ovarian endometrioid carcinoma (grade 2). (a) Adenocarcinoma infiltrating the submucosa of the rectum (arrowheads, H&E 40×). (b) Endometrioid carcinoma forming cribriform glands with a smooth luminal surface (H&E 100×). (c) Solid components are observed in part of the tumor (H&E 100×). (d) Some tumor cells exhibit squamous differentiation (H&E 400×) H&E: hematoxylin and eosin

Postoperatively, the specimen includes the rectum and the tumor, along with the anterior and posterior vaginal wall. The pathological diagnosis was recurrent ovarian endometrioid carcinoma grade 2 (Figure 4).

The margins were negative. The postoperative course was uneventful, with no recurrences for six months after surgery.

## Discussion

Secondary debulking surgery with complete resection has been reported to contribute to disease-free survival according to Gynecologic Oncology Group 213 (GOG213) and Descriptive Evaluation of Preoperative Selection Criteria for Operability in Recurrent Ovarian Cancer III (DESKTOP III) for recurrent ovarian cancer [2,3]. The GOG213 study reported that the hazard ratio for disease progression or death was not superior in the group receiving surgery plus chemotherapy. However, patients with platinum-sensitive recurrent ovarian cancer who underwent surgery plus chemotherapy experienced a longer progression-free survival compared to those who received chemotherapy alone, and complete resection proved beneficial. The DESKTOP Ⅲ trial reported that cytoreductive surgery followed by chemotherapy resulted in longer overall survival than chemotherapy alone.

Poly(ADP-ribose) polymerase (PARP) inhibitors such as olaparib have been reported to be effective in cases with BRCA mutations, such as initial maintenance therapy and recurrent maintenance therapy [10,11]. In addition, olaparib maintenance therapy has been approved in Japan for women with platinum-sensitive recurrent ovarian cancer who have a response to their most recent platinum-based regimen, regardless of BRCA mutation status. However, chemotherapy for recurrence after the use of PARP inhibitors is reported that the efficacy is poor [12]. Therefore, as in this case, a second debulking surgery was performed to treat the tumor, which progressed during the use of PARP inhibitors.

It is necessary to cooperate with doctors from other departments because of the possibility of invasion into other organs for recurrent ovarian cancer. In this case, rectal resection and intestinal resection were required, and after consultation with the colorectal surgeons regarding the surgical approach, the MIS was chosen. However, there is a lack of evidence regarding the efficacy of the MIS for recurrent ovarian cancer. Therefore, the patients should be treated by referral to a high-volume cancer center with surgeons who have intensive training in MIS [13].

In the operation for recurrent pelvic tumors, it is difficult to understand the anatomy surrounding the site of previous surgery. Therefore, the fluorescent ureteral NIRC for the real-time intraoperative visualization of the ureters is useful to see the running of the ureters. During MIS, the NIRC fluorescent ureteral catheter can be visualized only by switching the endoscopic mode to fluorescence observation mode with the near-infrared ray using the button at hand. In addition, the NIRC fluorescent ureteral catheter can easily be inserted like a normal ureteral stent before surgery.

This catheter is very useful because it allows the dissection of the ureters from the anterior vaginal wall and the tumor. Without this catheter, the ureters cannot be dissected safely, and anterior pelvic resection was needed. We believe that the use of the NIRC fluorescent ureteral catheter will contribute to the MIS and to reducing the risk of conversion to open surgery.

## Conclusions

Pelvic exenteration is one of the operations for central pelvic recurrence. However, even a well-trained, educated, and highly skilled gynecologist could deal with recurrent tumors in laparoscopic surgery, preserving the ureter and the bladder due to the real-time intraoperative visualization of the ureters. We have encountered a case involving laparoscopic resection of a recurrent tumor at the vaginal stump, along with dissection of the bladder and ureters from the anterior vaginal wall. This was done using a fluorescent ureteral NIRC as a guide following primary surgery for ovarian cancer.
